# Ankle joint flexibility affects undulatory underwater swimming speed

**DOI:** 10.3389/fspor.2022.948034

**Published:** 2022-08-10

**Authors:** Jessica Kuhn, Kirsten Legerlotz

**Affiliations:** Institute of Sport Sciences, Humboldt-Universität zu Berlin, Berlin, Germany

**Keywords:** undulatory underwater swimming, dolphin kick, swimming performance, ankle joint flexibility, plantar flexion, elite swimmers

## Abstract

The movement of undulatory underwater swimming (UUS), a swimming technique adapted from whales, is mainly limited by human anatomy. A greater ankle joint flexibility could improve the imitation of the whale's flap of the fin and therefore enhance USS performance. The aim of this study was to investigate the impact of ankle joint flexibility on swimming velocity and kick efficiency during UUS by comparing kinematics of swimming trials with reduced, normal, and enhanced maximum angles of plantar flexion. Ten well trained swimmers (5m and 5f; 22 ± 4years; 177 ± 7cm; 74 ± 15kg), performed multiple trials of UUS with normal, restricted, and increased ankle joint flexibility on two separate days in randomized order. Kick frequency was controlled by a metronome. Plantar flexion (PF) was restricted by tape application on both feet and increased by passive-dynamic stretching. All trials were filmed. Kinematics were obtained with two-dimensional motion analysis. Tape application restricted maximum PF by 10.42% while stretching increased PF by 6.87% compared to normal PF. Swimming velocity and kick efficiency significantly decreased during swimming with restricted PF (1.13 ± 0.13m^*^s^−1^; 0.69 ± 0.09m) compared to normal (1.20 ± 0.14 m^*^s^−1^; 0.72 ± 0.10m) and increased (1.22 ± 0.15m^*^s^−1^; 0.73 ± 0.10m) PF. Swimming velocity and kick efficiency did not differ between normal and increased PF. Body height normalized swimming velocity correlated significantly with PF angle (*r* = 0.538). The results suggest that UUS velocity is affected by impaired PF. Particularly swimmers with low or average maximum PF angles may benefit from a long-term ankle joint flexibility program to improve their UUS performance.

## Introduction

In competitive swimming races, success and failure are often discriminated by milliseconds only (McCullough et al., 2009; Yuan et al., 2019). As a result, biomechanical characteristics affecting swimming stroke efficiency must be identified and optimized to improve the athlete's performances even by marginal gains. Since undulatory underwater swimming (UUS) is often faster than the main swimming strokes (Bissig et al., 2004; Ungerechts et al., 2009; Schneider, 2012), improving dolphin kick efficiency has the potential to improve overall competitive swimming times (Nakashima, 2009; Gonjo and Olstad, 2020). The more streamlined body posture (Schneider, 2012; Zamparo et al., 2012), as well as the smaller up to non-existing wave resistance underneath the water surface allow to maintain the gliding speed after start and turns as long as possible and to reduce the deceleration during the diving phase (Zamparo et al., 2012). Although the optimal distance traveled underwater seems to be individually different and depends on the following swimming stroke (Veiga and Roig, 2015; Veiga et al., 2016; Morais et al., 2019), studies have shown that faster swimmers had longer UUS distances (Veiga et al., 2014) and UUS can have a positive impact on swimming velocity and stroking length on start and turn segments (Veiga and Roig, 2016) as well as total race times (Morais et al., 2019). Thus, the permitted diving distance of 15 m (Fédération internationale de Natation, 2017), which equates to 30% of a long course and 60% of a short course, provides an opportunity to improve overall competitive swimming times by enhancing UUS performance.

To maximize the speed of UUS, the optimized movement must be adapted to human anatomy (Hochstein and Blickhan, 2014). Since the human body has only a few joints that can execute the undulatory movement (hip, knee and ankle), the smooth transition of the body wave is highly limited and the propulsion effect is quite low compared to whales with a larger number of separate joints (Von Loebbecke et al., 2009a,b). However, a greater ankle joint flexibility which allows increased plantar flexion may improve the dolphin kick performance as the greatest propulsion is generated with the kicking movement of the feet (Von Loebbecke et al., 2009b). More flexible ankle joints could thus superiorly imitate the efficient kicking movement of a fin (Reischle, 1988; Wick, 2013). The displacement of water during the down kick would be directed rather backwards than downwards, so there would be a higher propulsion with the same power efficiency (McCullough et al., 2009; Hochstein et al., 2010; Schneider, 2012; Séhel, 2016). Furthermore, the greater range of motion could increase the flipping movement of the feet which would enhance the usable propulsive momentum by faster reversion of the vortices (Strass et al., 2002; Ungerechts et al., 2009). Additionally, a greater ankle joint flexibility could result in a more harmonized and energy-efficient undulatory movement (Hahn, 2013). This could enhance the kicking frequency which, in turn, is positively correlated to swimming speed (Arellano et al., 2003; McCullough et al., 2009). Therefore, elite as well as recreational athletes could improve their swimming performance in different strokes *via* more efficient UUS by enhancing their ankle joint flexibility.

Previous studies of UUS mainly investigated kinematic key parameters as kicking frequency and kick amplitude (Arellano et al., 2002; Connaboy et al., 2009; Cohen et al., 2012; Yamakawa et al., 2017) or the underlying hydrodynamics (Arellano et al., 2002; Connaboy et al., 2009; Von Loebbecke et al., 2009b). The potential benefit of more flexible ankle joints on kicking efficiency and swimming velocity is often mentioned but rarely directly tested. Only a few studies investigated the effect of the ankle joint flexibility on swimming velocity during UUS (Sugimoto et al., 2008; Willems et al., 2014; Connaboy et al., 2016; Shimojo et al., 2019; Wadrzyk et al., 2019). Different methodological approaches (e. g., different swimming distances, different number of analyzed swimming trials and swimming cycles per trial, different magnitude of ankle joint flexibility restriction, dimension of filming and analysis) complicate the direct comparison of the results. A restriction of the plantar flexion (PF) angle by tape application consistently decreased swimming velocity, however, it remained unclear if an increase in the range of ankle movement would enhance the swimming velocity.

The aim of this study was to investigate the impact of ankle joint flexibility on swimming velocity and kick efficiency during UUS by comparing kinematics of swimming trials with different maximum angles of plantar flexion. We hypothesized that a greater ankle joint flexibility (maximum PF angle) is associated with a greater swimming velocity and kick efficiency.

## Materials and methods

### Subjects

Five male and five female swimmers (age: 22.00 ± 4.19 years, height: 176.90 ± 6.64 cm, weight: 74.20 ± 15.11 kg, training experience: 15.7 ± 4.0 years) were tested within this study. Swimmers who reported former ankle surgery or structural ankle injuries were excluded from participation in this study. The study was reviewed and approved by the Ethics Committee of the Faculty of Humanities and Social Sciences of the Humboldt-Universität zu Berlin. The participants provided their written informed consent to participate in this study.

### Experimental setup

Each participant got tested on two separate days at an interval of a week. Anthropometric data (body height and weight) and personal data (age, sex, and training experience) were recorded before starting the swimming trials.

Swimming trials were performed in an indoor swimming pool (8 × 25 m, water temperature: 28 °C, water depth: 1.80 m). Swimmers maintained a depth of ~0.8 m while performing UUS. The participants were instructed to use the push off from the wall only to obtain the correct water depth, and to generate the swimming speed by undulatory swimming only. All trials were filmed, and kinematics were obtained by two-dimensional motion analysis.

### Standardization of kicking frequency

After independently warming up for 20 min including UUS, the participants performed three trials (15 m) of underwater dolphin kicks in maximum speed to determine their maximum kicking frequency. Between the trials they had a resting time of 5 min. The duration of three kick cycles were used to calculate the kicking frequency. The highest frequency of the three trials was set as individual maximum (100%).

The following trials were performed with submaximal effort (80% of individual maximum kicking frequency). The submaximal effort should provide an individual competition-like intensity without risking to much muscle fatigue. The constant kicking frequency during all swimming trials should also ensure a consistent power output and prevent an impact of kicking frequency on UUS kinematics. Shimojo et al. (2014) showed no significant difference of kick efficiency (Strouhal number and Froude-efficiency) during swimming with submaximal kicking frequency (85%) compared to swimming with maximum kicking frequency. The calculated velocity was set by a waterproof metronome device (FINIS^®^ Tempo Trainer Pro) which was clipped onto the swimming goggle. The synchronization of the kicking frequency to a metronome device has previously been shown to have no impact on kinematics, movement patterns and muscle activity of the lower extremities during UUS (Yamakawa et al., 2017). The swimmers performed three familiarization trials of 15 m with underwater dolphin kicks trying to synchronize their kicking frequency to the beat of the metronome device.

### Swimming trials

The familiarization trials were followed by three trials of swimming with normal, restricted, and increased ankle joint flexibility, respectively. The participants were asked to swim as fast as possible while maintaining the set kick frequency of the metronome device.

The following conditions were tested and compared regarding different kinematic parameters:

1. Normal PF angle: participants swam with their natural ankle joint flexibility,
2. Restricted PF angle: plantar flexion was restricted by tape application on both feet by approximately 10% before swimming,
3. Increased PF angle: plantar flexion was increased by passive-dynamic stretching before swimming.

The condition “normal PF angle” was tested first on both test days. The order of the remaining conditions was individually randomized. Accordingly, there were two possible orders:

1. 3 x Normal – 3 x Restricted – 3 x Increased,
2. 3 x Normal – 3 x Increased – 3 x Restricted.

On the second testing day the order of the last two conditions was reversed compared to the first testing day.

There were 3 min rest between trials and 10 min rest between sets. The application of the tape and the passive-dynamic stretching was performed during that resting time.

### Taping

The amount of restriction was aimed to be high enough to produce measurable effects on swimming velocity and kick efficiency and low enough to prohibit unwanted effects on swimming technique. Considering previous studies which used either 30 or 4% (Willems et al., 2014; Shimojo et al., 2019), we aimed for a restriction in between of 10% of maximum plantar flexion.

Right before the swimming trials, the active PF angle was measured with a goniometer using the neutral zero method (Freiherr von Salis-Soglio, 2015). A waterproof elastic tape was used to restrict PF and all swimmers got taped by the same person (JK). The feet were held in a position of 80% of maximum PF angle while applying the tape as tightly as possible. The remaining restriction was supposed to result in 90% of maximum PF angle. After tape application, the active maximum PF angle was measured again to verify, that the sought PF restriction was achieved. If the tape loosened partially from the feet during swimming trials the tape application was renewed during the following resting time.

### Stretching

Immediately before the swimming trials with increased PF a passive-dynamic stretching of the ankle joints was performed. Swimmers lay on the ground with straightened legs while the researcher (JK) moved the feet from maximum plantar flexion to maximum dorsiflexion within 5 s. This stretching was performed for 60 s and paused for 30 s. Before the first trial, the stretching was repeated three times. During the resting time between trials, the stretching was performed once to maintain the acute stretching effect. After every finished stretching session, the maximum active PF angle was measured. The plantar flexion angle increased on average by 6.87%. To counteract possibly reduced muscle activity after stretching, the swimmers performed three hops before each swimming trial.

### Motion analysis

**Figure 1:**
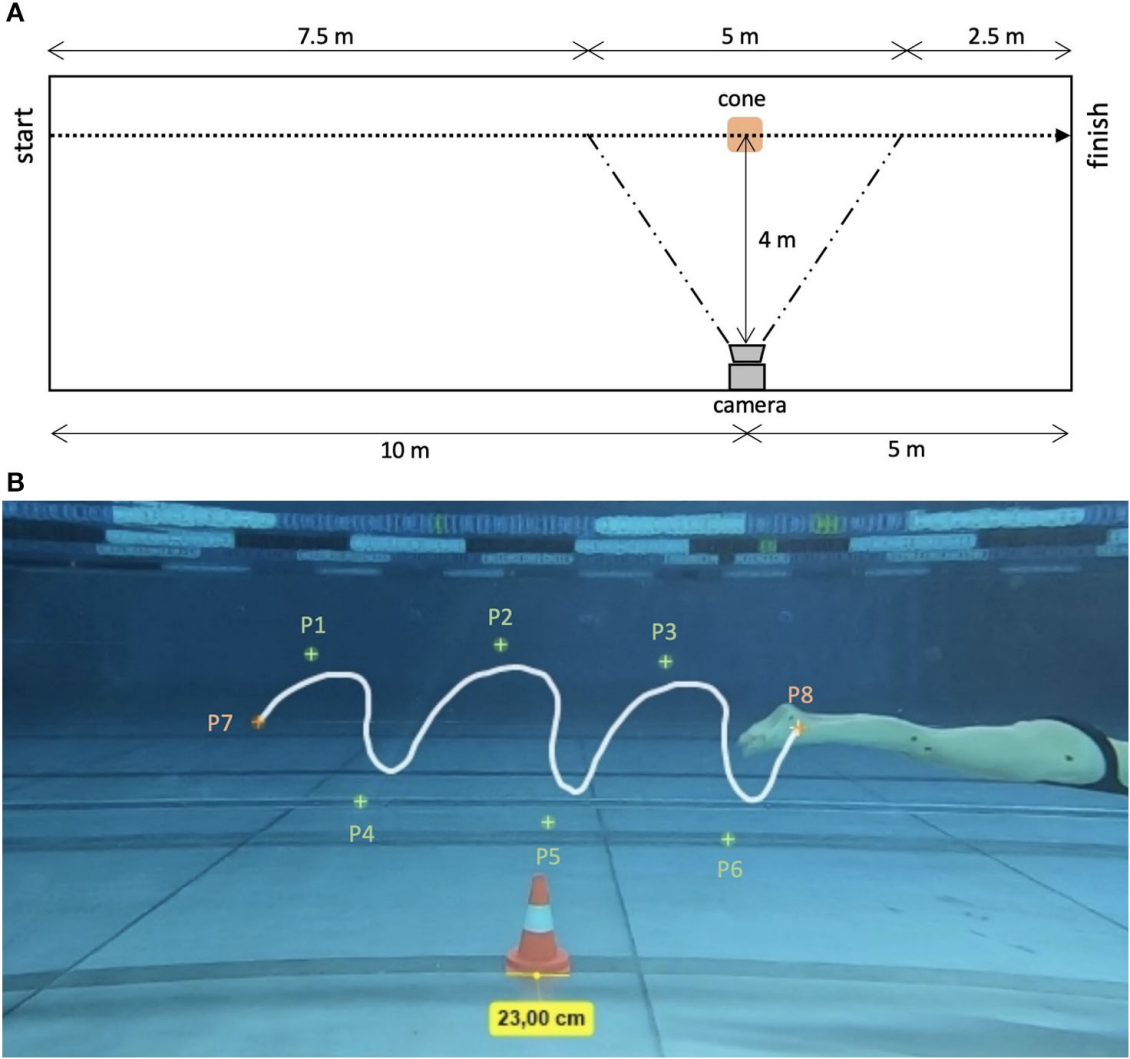
Experimental set-up **(A)** and motion analysis of swimming trial by tracking bony landmarks **(B)**. P1–P3 and P4–P6 mark the highest and lowest points of the fifth toe during kicking cycles. P7 and P8 mark the start and end points of three kicking cycles.

UUS trials of the participants were filmed with an underwater video camera (60 frames per second; GoPro HERO7, GoPro Inc., San Mateo, USA) which was positioned 0.6 m below water surface and 10 m away from the starting point (perpendicular to the swimming direction). The camera was attached to a bar that was pressed against the wall of the pool to ensure a stable camera positioning while filming. The distance between camera and swimmers was 4 m. The recorded area of swimming was from 7.5 m to 12 m after push-off from the wall. A cone was placed 10 m from the starting point (push-off). Its width was used as reference for calibration of the swimming distance in the motion analysis program (see Figure 1).

For motion analysis, six anatomical landmarks were marked with a waterproof pen on the lateral right side of the swimmer's bodies: trochanter major (hip), epicondylus lateralis femoris (knee), caput fibulae (knee), malleolus lateralis (ankle), calcaneus (heel) and caput ossis metatarsalis V (toe). The recorded videos were uploaded to a motion analysis program (Kinovea version 0.9.4) and landmarks of the swimmer's bodies were manually digitized (see Figure 1). A recent study reported that the Kinovea software is a valid and reliable tool that is able to measure accurately at distances up to 5 m from the object and at an angle range of 90–45° (Puig-Divi et al., 2019).

The following kinematic variables were measured, respectively, calculated:

1. kicking frequency [Hz]: number of finished kicking cycles divided by duration of swimming,
2. kick amplitude [m]: vertical distance between highest and lowest position of the fifth toe during kick cycles,
3. horizontal swimming velocity[m^*^s^−1^]: swimming distance divided by swimming duration,
4. kick efficiency [m/kick]: horizontal swimming velocity divided by kicking frequency, and
5. minimum knee flexion angle: α [°]: minimum angle between femur and fibula during the down kick.

Three kick cycles of each trial were used to calculate the mean of each variable. For statistical comparison of the three different PF conditions, means were calculated of three trials first and of both testing days afterwards.

### Statistical analysis

Statistical analysis was performed using IBM SPSS (version 27). Shapiro-Wilk-tests were used for assessment of normal distribution. All kinematic variables were statistically normally distributed except minimum knee flexion angle during swimming with increased PF angle [*W*_(10)_ = 0.83, *p* = 0.036, *n* = 10]. *T*-tests for dependent samples were used to compare the kinematics of the two separate testing days. An analysis of variance (ANOVA) for repeated measurements was applied to compare kinematics of the different swimming conditions for all variables, as it is robust against violations of the normal distribution. In case of a significant difference, a Bonferroni *post-hoc* analysis was conducted. The effect size *f* was evaluated as small (0.10–0.24), moderate (0.25–0.39) and large (>0.40) (Cohen, 1988). Pearson correlations *r* were used to determine the correlation between maximum PF angle and each kinematic parameter. Classification was made regarding the minimum levels of *r*: small (±0.1), moderate (±0.3) and large (±0.5). The level of significance was set at *p* < 0.05.

## Results

There was no significant effect of testing day on UUS kinematics, thus the mean of both days was calculated and used for analysis of each tested condition (PF angle).

Maximum PF angles were significantly lower during swimming with restricted PF angle compared to swimming with normal or increased PF angle (Table 1). The effect size was evaluated as large (*f* = 4.36).

**Table 1:** Kinematic variables of USS with restricted, normal, and increased plantar flexion (mean±SD).

**Kinematic variable**	**Ankle joint flexibility**		
	**Restricted**	**Normal**	**Increased**
Maximum plantar flexion	57.5 ± 3.51	64.2 ± 3.94*	68.6 ± 4.76*^,^ ^#^
angle [°]			
Minimum knee flexion angle [°]	107.9 ± 7.74	108.4 ± 7.97	109.2 ± 7.89*
Kicking frequency [Hz]	1.66 ± 0.17	1.68 ± 0.18	1.67 ± 0.19
Kick amplitude [m]	0.64 ± 0.08	0.65 ± 0.08	0.66 ± 0.09

* Significantly different to restricted PF angle,

# significantly different to normal PF angles.

Kicking frequency, as set by the metronome, did not differ between test conditions as planned (Table 1).

**Figure 2:**
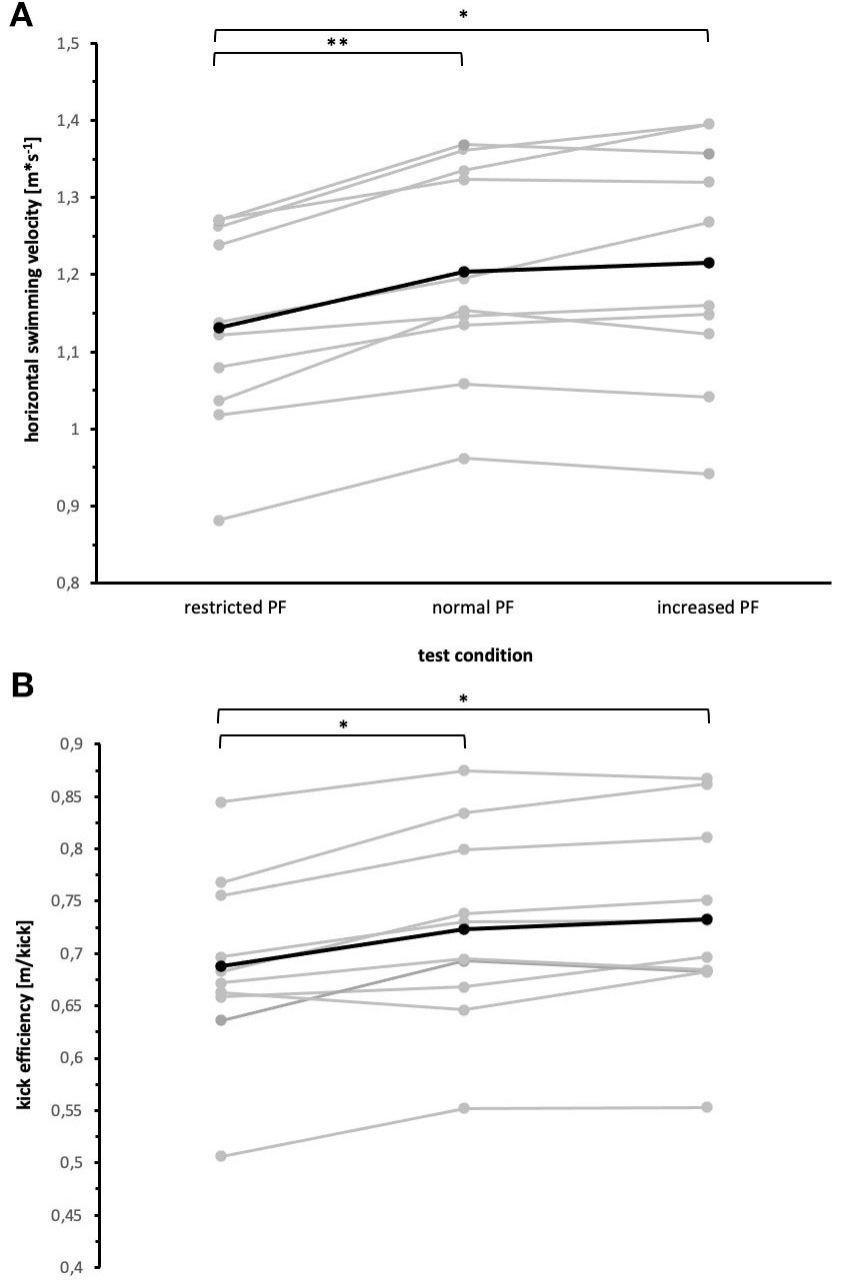
Effect of increased, normal, and restricted plantar flexion angle on horizontal swimming velocity **(A)** and kick efficiency **(B)**. Gray lines represent individual swimmers while the black line represents the mean value. *p < 0.05, **p < 0.001.

Kick efficiency and horizontal swimming velocity were significantly smaller during swimming with restricted PF angle compared to swimming with normal and increased PF (Figure 2). The effect size was evaluated as large (kick efficiency *f* = 1.54; swimming velocity *f* = 1.82). There were no significant differences regarding kick efficiency and horizontal swimming velocity between swimming with normal and increased PF.

Regarding kick amplitude, the ANOVA for repeated measurements indicated a significant difference in kick amplitude between tested conditions [*F*_(2,18)_ = 3.74, *p* = 0.044, $\eta_{p}^{2}$ = 0.29]. Mean values for kick amplitude were highest during swimming with increased PF angle followed by swimming with normal PF angle and restricted PF angle (Table 1). However, Bonferroni corrected paired comparisons did not reveal significant differences between conditions.

Minimum knee flexion angle was significantly smaller during swimming with restricted PF compared to swimming with increased PF (Table 1). The effect size was evaluated as large (*f* = 0.79). There was no significant difference of minimum knee flexion angle between swimming with normal PF and restricted PF as well as between swimming with normal and increased PF.

**Figure 3:**
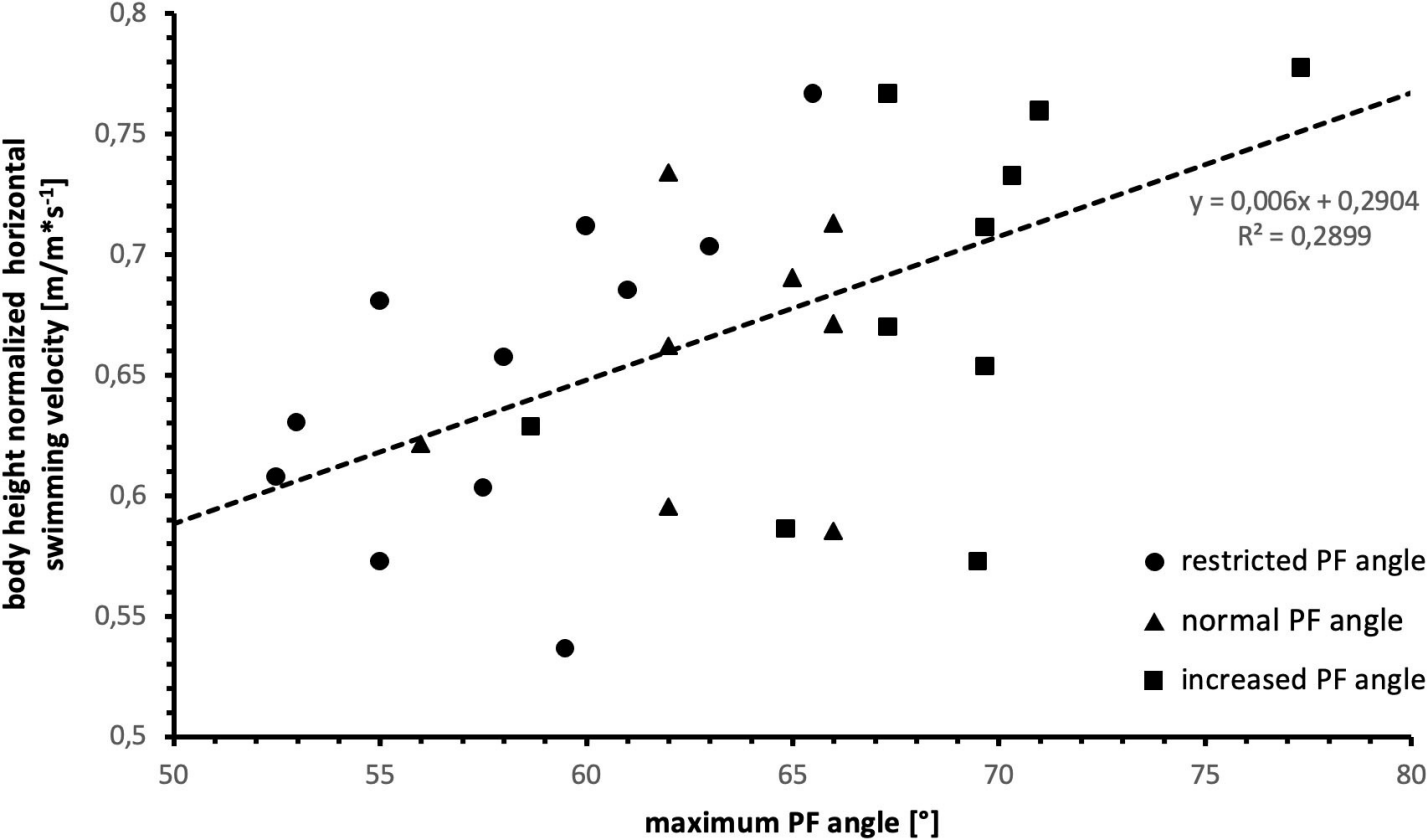
Relationship between body height normalized swimming velocity and maximum PF angle.

Horizontal swimming velocity significantly correlated with body height in all tested conditions (restricted PF: *r* = 0.77, *p* = 0.010; normal PF: *r* = 0.66, *p* = 0.038; increased PF: *r* = 0.64, *p* = 0.046). Therefore, to account for interpersonal differences in body morphology affecting swimming performance and to analyze the effect of maximum PF angle irrespective of body morphology, swimming velocity was normalized to body height for further analysis. Body height normalized swimming velocity significantly correlated with maximum PF angle (*r* = 0.538, *p* = 0.002) which is shown in Figure 3.

There were no significant correlations between maximum PF angle and other kinematic variables.

## Discussion

To our knowledge this is the first study that investigated the impact of both restricted as well as increased plantar flexion angles on UUS kinematics. In agreement with our hypothesis, swimming velocity and kick efficiency were affected by ankle joint flexibility. However, it was particularly the restriction of plantar flexion that was negatively affected, while the increase in plantar flexion did not further enhance swimming velocity or kick efficiency.

The negative effect of PF restriction on swimming velocity seems to be at least partially dose dependent. A study on the effect of ankle flexibility on dolphin kick performance in competitive swimmers, which restricted PF by tape application by 30% (Willems et al., 2014), has led to a 19.5% reduction in swimming velocity, while in our study PF restriction was smaller (10%) which has led to a smaller reduction in swimming velocity of 5.8%. In accordance, a small PF restriction of 4% in a study on ankle joint flexibility in UUS (Shimojo et al., 2019) resulted in a small reduction of swimming velocity of 4.7%.

Regarding the reason for the effect of PF restriction on swimming velocity, it has been suggested (Willems et al., 2014) that the area of the feet, which shifts the water backwards and generates the propulsive impulse, decreases with the restriction of the PF angle. Consequently, a higher knee flexion during the down kick has been observed which may be a compensatory strategy for restricted PF flexibility (Willems et al., 2014). The limited vortex generation due to restricted PF and the potentially greater frontal drag due to higher knee flexion were suggested to be the reason for the decreased swimming velocity. Previous studies also described a negative correlation of knee flexion and swimming velocity because of the higher frontal water resistance [*r* = −0.70 (Arellano et al., 2003); *r* = −0.53 (Wadrzyk et al., 2017)]. Our results point in the same direction as the reduction of swimming velocity with restricted PF angle was similarly accompanied by greater knee flexion when compared to swimming with increased PF angle (+1.2%). It is conceivable, that smaller reductions in PF lead to smaller or negligible changes in knee flexion. This is supported by the finding that a 6% reduction in PF (Wadrzyk et al., 2019) did not result in any significant changes in knee flexion angles.

It has also been suggested (Sugimoto et al., 2008; Cohen et al., 2012) that a limited inversion of the feet may cause a reduction in swimming velocity. In the present study, the measurement of the foot inversion angle was not possible due to the lateral and two-dimensional nature of the video analysis. Thus, we cannot test this assumption with our data. A previous study (Matsuda et al., 2021) detected no significant relationship between ankle inversion ROM and UUS velocity. However, this study analyzed the correlation between ankle inversion ROM and UUS velocity in fast and less fast swimmers and thus described interpersonal differences. Those interpersonal differences in UUS velocity can be affected by a huge variety of variables not allowing to conclude on the effect of ankle inversion ROM on UUS velocity within one person. While the combination of maximum plantar flexion and inversion may be important for a perfect propulsion during UUS, we are confident that there is a causal relationship between PF restriction and decreased swimming velocity in our study. In agreement with the literature, the restricted maximum PF angle could have caused a greater frontal drag (Ungerechts et al., 2009). Additionally, the smaller range of motion could have resulted in a limited flipping action of the feet during the down kick so both the size and the rotation velocity of the generated vortices could have been reduced, which, in turn, would have decreased the swimming velocity and kick efficiency. Also, the tape material could have changed the streaming characteristics of the water as well as the vortex generation which could have contributed to the decreased swimming velocity and kick efficiency. However, the extent of this possible impact cannot be determined yet.

In contrast to the negative effect of PF restriction on swimming velocity, it seems that an increase in PF by stretching intervention does not further enhance swimming velocity. It is conceivable, that the increase in PF angle compared to the normal condition may have been too small to result in significant changes in swimming velocity and kick efficiency. It is also possible that other three-dimensional movements of the lower limbs are crucial to achieve higher UUS velocity, as it has been shown that e. g., the peak angular velocities of hip internal and external rotation were significantly correlated with UUS velocity (Matsuda et al., 2021). However, body-height normalized swimming velocity highly significantly correlated with maximum PF angle in the present study which indicates that the ankle joint flexibility affects swimming velocity. This result contrasts with the findings of Willems et al. (2014) who did not find a significant correlation between swimming velocity and PF angle. They assumed that the ankle joint flexibility is a neglectable factor regarding swimming velocity compared to other determinants like muscle power or water drag. While other determinants may affect swimming velocity to a greater extend, we suppose that PF angle has at least a small impact on swimming velocity, which may decline with increasing ankle flexibility. Thus, there can be an optimal condition of plantar flexion flexibility beyond which no further gain in swimming velocity and kick efficiency is realized. Furthermore, the normal PF angles of our participants were with 50° highly above average. It is possible that in swimmers with lower initial values of PF angle a larger effect on swimming velocity and kick efficiency may have been measurable which needs to be confirmed in future studies. However, especially in longer races even small improvements of kick efficiency can have a particular impact on overall performance as a higher kick efficiency can result in lower energy cost and therefore faster racing times (Zamparo et al., 2020). Over a 400 m short course race a time improvement of ~2 s can be calculated based on the within our study detected improvements in kick efficiency and swimming velocity with increased PF (compared to normal PF), when swimming with an intensity of 80% and assuming an UUS distance of 10 m excluding start and turn push offs.

Considering the presented results, it is conceivable that particularly athletes with low or average maximum plantar flexion angles could benefit from of a long-term ankle joint flexibility program to improve their UUS and overall performance.

Although the study was conceived and performed with care to obtain objective, valid and reliable data, there are some points to discuss that may limit the interpretation of the results. The present study investigated acute effects of ankle joint flexibility changes only. However, long-term increases in plantar flexion flexibility may differently affect UUS velocity. For instance, we observed a higher knee flexion, possibly resulting from an acute compensatory mechanism in response to restricted PF but long-term impacts of limited PF on knee flexion cannot be derived from the presented results and may differ from acute effects. Moreover, the elastic material of the tape may not have fully restricted the PF angle during swimming because of the high passive forces underwater. A non-elastic tape could have reduced this potential discrepancy between maximum PF angle on land and during swimming. However, when applying a non-elastic tape the participants of Shimojo et al. (2019) reported pain during swimming. Furthermore, pilot-trials of the present study demonstrated that a non-elastic tape was not waterproof, so the tape got loosened during the swimming trials and PF angle was no longer restricted. For this reason, a waterproof elastic tape was used in the present study and was sticked as tightly as possible onto the skin. In addition, exact measurement of PF angle and inversion during swimming was not feasible due to two-dimensional video analysis and combined movement of PF and inversion during down kick. Thus, future studies should consider a 3D movement analysis to capture foot inversion in addition to PF. Besides, an underwater camera was used to film the swimming trials. While a linear field of view was set, there was a slight distortion at the outer frame of the video. To counteract this effect, analyzed swimming trials were always in the center of the video. Lastly, the study size may have been too small to find small yet significant changes of e. g., kick amplitude and a resulting impact on UUS performance as well as gender-specific effects.

Further research is necessary to determine the magnitude of the impact of ankle joint flexibility on swimming velocity and kick efficiency as well as the threshold level of PF angle upon which swimming performance does not further improve. Kinematics between swimming with normal and increased PF angles should be tested and compared particularly in swimmers with impaired ankle flexibility to observe the effects of increased PF angle on UUS performance.

## Implications for practice

Since success in competitive swimming races is often determined by milliseconds, factors affecting swimming stroke efficiency must be identified and optimized to improve the athlete's performances even by marginal gains. As reduced ankle joint flexibility impairs UUS velocity, we recommend that particularly swimmers with low or average PF angles should consider implementing ankle joint flexibility exercises in their training regime to improve their UUS performance.

## Data availability statement

The raw data supporting the conclusions of this article will be made available by the authors, without undue reservation.

## Ethics statement

The studies involving human participants were reviewed and approved by the Ethics Committee of the Faculty of Humanities and Social Sciences of the Humboldt-Universität zu Berlin. The patients/participants provided their written informed consent to participate in this study.

## Author contributions

JK conceived the study and collected the data, performed the analysis, and wrote the first version of the manuscript. JK and KL designed the study and discussed the results and contributed to the final version of the manuscript. KL supervised data collection and analysis and revised the manuscript. All authors contributed to the article and approved the submitted version.

## Funding

The publication of this article was funded by Humboldt-Universität zu Berlin. The funder had no role in study design, data collection, analysis, decision to publish, or manuscript preparation. The article processing charge was funded by the Deutsche Forschungsgemeinschaft (DFG, German Research Foundation) - 491192747 and the Open Access Publication Fund of Humboldt-Universität zu Berlin.

## Conflict of interest

The authors declare that the research was conducted in the absence of any commercial or financial relationships that could be construed as a potential conflict of interest.

## Publisher's note

All claims expressed in this article are solely those of the authors and do not necessarily represent those of their affiliated organizations, or those of the publisher, the editors and the reviewers. Any product that may be evaluated in this article, or claim that may be made by its manufacturer, is not guaranteed or endorsed by the publisher.
